# Serum 25-hydroxyvitamin D and cognitive performance in hospitalized older adults: a cross-sectional study from Central Poland

**DOI:** 10.3389/fnut.2026.1887375

**Published:** 2026-08-13

**Authors:** Ganna Kravchenko, Serena S. Stephenson, Julia Dmuchowska, Tomasz Kostka, Bartłomiej K. Sołtysik

**Affiliations:** Department of Geriatrics, Healthy Ageing Research Centre (HARC), Central Teaching Hospital of the Medical University of Lodz, Łódź, Poland

**Keywords:** 25-hydroxyvitamin D, cognitive performance, dementia, geriatrics, hospitalized older adults, nutritional status, vitamin D

## Abstract

**Introduction:**

Dementia constitutes a major and rapidly growing public health challenge worldwide. The identification of its risk factors and potential contributors has therefore become a major focus of research. This study aimed to investigate the association between serum 25(OH)D concentration and cognitive performance, as well as the odds of cognitive impairment and documented dementia in a hospitalized geriatric population.

**Materials and methods:**

This cross-sectional study included 1,539 hospitalized individuals aged ≥60 years from Central Poland. Cognitive performance was assessed using the Mini-Mental State Examination (MMSE) and the Clock Drawing Test (CDT). The age- and education-adjusted MMSE score was used in the statistical analyses. The test-based adverse cognitive outcomes were defined separately as MMSE <24, based on the age- and education-adjusted MMSE score, and CDT < 6. Information on documented dementia was obtained from medical records. Univariate analysis was performed using Mann–Whitney *U* test for continuous variables and chi-square test for categorical variables. Multivariable logistic regression models were used to assess the associations between serum 25(OH)D concentration and MMSE < 24, CDT < 6, and documented dementia, with adjustment for age, sex, BMI, albumin concentration, depressive symptoms, and history of stroke.

**Results:**

Vitamin D deficiency (<20 ng/mL) was present in 53.3% of participants, and only 27.6% had concentrations ≥30 ng/mL. Higher serum 25(OH)D concentration was associated with lower odds of MMSE < 24 (OR = 0.984; 95% CI: 0.973–0.994; *p* = 0.001), CDT < 6 (OR = 0.988; 95% CI: 0.978–0.998; *p* = 0.018), and documented dementia (OR = 0.980; 95% CI: 0.970–0.990; *p* < 0.001). These estimates correspond to approximately 11–18% lower odds of adverse cognitive outcomes per 10 ng/mL increase in serum 25(OH)D. Older age, higher depressive symptom burden, and history of stroke were associated with worse cognitive outcomes, whereas higher albumin concentration was associated with better cognitive performance.

**Conclusion:**

Higher serum 25(OH)D concentration was associated with better cognitive performance and lower odds of cognitive impairment and documented dementia in hospitalized older adults. These findings should be interpreted as observational associations. Due to the cross-sectional design, causality cannot be inferred, and prospective studies are warranted.

## 1 Introduction

Dementia represents a major and growing public health challenge worldwide. In 2019, approximately 57 million people were living with dementia, and this number is expected to increase substantially with population ageing (1). Low serum 25-hydroxyvitamin D [25(OH)D] is highly prevalent in older adults and is recognized as an important geriatric problem (2). Large epidemiological studies and recent meta-analyses indicate that a substantial proportion of older individuals have deficient or insufficient vitamin D levels, with advanced age itself being an independent risk factor for low 25(OH)D status (3, 4). In geriatric populations, reduced cutaneous synthesis due to limited sun exposure, inadequate dietary intake, multimorbidity, and polypharmacy frequently contribute to impaired vitamin D metabolism (5, 6). These factors are particularly pronounced in hospitalized older patients, who are characterized by high disease burden and increased nutritional risk (7).

Several biological mechanisms may link 25(OH)D deficiency with neurodegeneration and cognitive decline. These include modulation of neurotrophic factors, regulation of neuronal calcium homeostasis, attenuation of neuroinflammation, and potential effects on cerebrovascular function and amyloid-β clearance (8). Through these pathways, inadequate vitamin D status may influence both Alzheimer’s disease and vascular cognitive impairment. Consistent with this biological plausibility, numerous observational studies have reported associations between lower serum 25(OH)D concentrations and poorer cognitive performance or increased risk of cognitive decline and dementia (9–11). However, evidence from randomized and interventional studies remains limited and inconsistent, and causality has not been established.

Importantly, most previous studies have focused on relatively healthy, community-dwelling adults aged 60–70 years, often excluding individuals with severe comorbidity, malnutrition, or functional impairment (11, 12). Consequently, their findings may not be directly applicable to hospitalized geriatric patients, who represent a particularly vulnerable group with advanced age, high multimorbidity, inflammatory burden, and frequent nutritional deficiencies. Moreover, few hospital-based studies have simultaneously accounted for markers of nutritional status and systemic inflammation when examining the relationship between vitamin D and cognitive function.

In this context, the present study aimed to investigate the association between serum 25(OH)D concentration and cognitive performance assessed by the Mini-Mental State Examination (MMSE) and the Clock Drawing Test (CDT) in a hospitalized geriatric population. Additionally, we examined the relationship between vitamin D status and the odds of documented dementia. We hypothesized that lower serum 25(OH)D concentrations would be associated with poorer cognitive performance and higher odds of documented dementia, even after adjustment for nutritional status, depressive symptoms, and major clinical covariates.

## 2 Materials and methods

### 2.1 Patients

**Figure 1:**
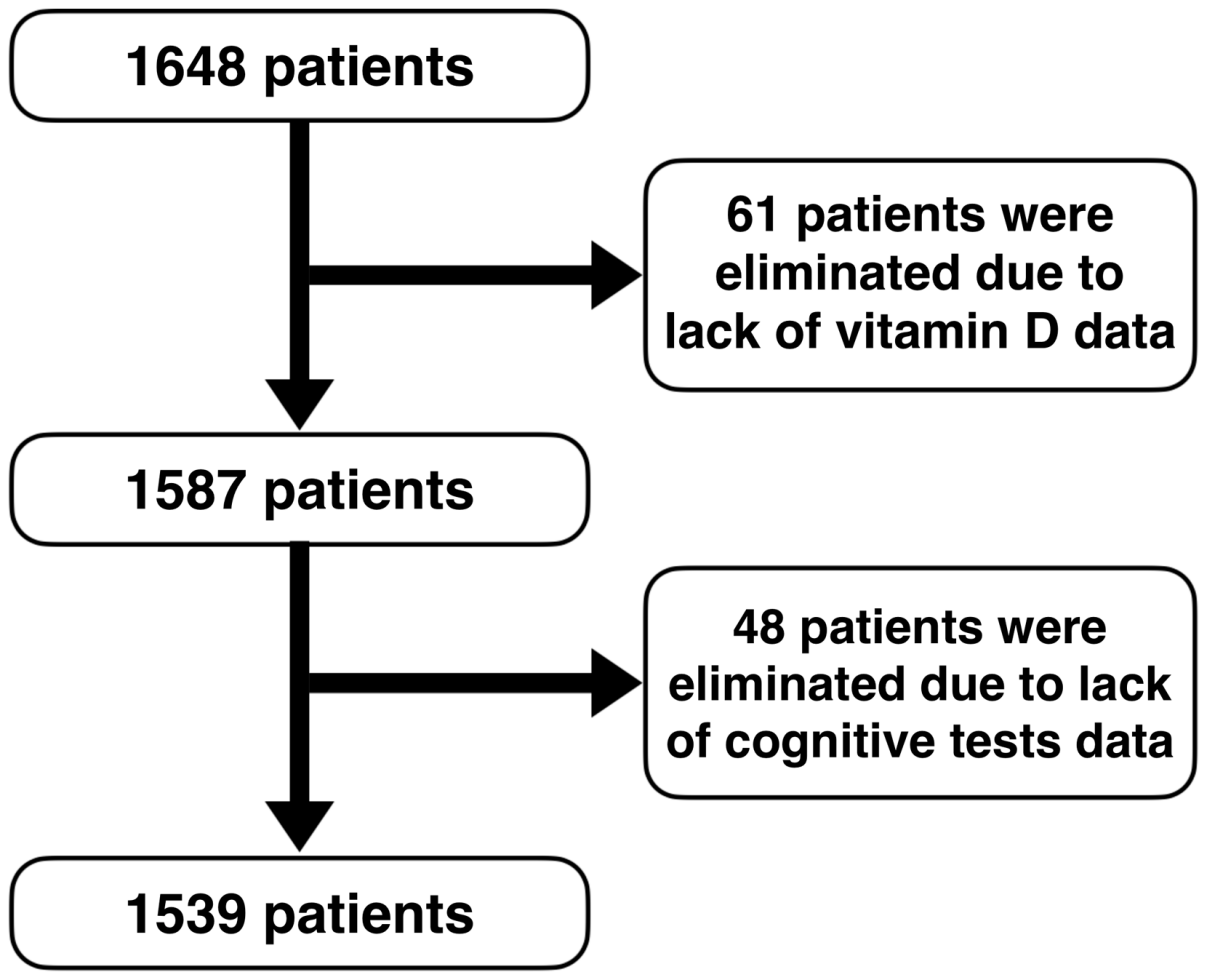
Flowchart of participant selection.

This was a hospital-based, cross-sectional observational study conducted in a geriatric inpatient population. Patients were enrolled consecutively between October 2022 and March 2024 in the Department of Geriatrics, Medical University of Lodz, Poland. The inclusion criteria were age ≥60 years, ability to communicate verbally, and provision of informed consent. Patients with severe aphasia, acute delirium, or other conditions precluding reliable cognitive assessment were not included. Of the 1,648 initially screened patients (Figure 1), 61 had missing 25(OH)D measurements and 48 had missing cognitive test results and were excluded (final *N* = 1,539; 1,057 women and 482 men). A comparison of included and excluded participants is presented in Supplementary Table S1. Analyses were performed using complete cases; therefore, no data imputation was applied. Patients were enrolled consecutively throughout the study period, which reduced potential seasonal selection bias; however, month of blood sampling was not included in the primary statistical models.

### 2.2 Anamnesis

Upon admission, a detailed examination of the patient’s medical history was performed. The presence of arterial hypertension (HT), diabetes mellitus type 2 (DM), lipid disorders, coronary artery disease (CAD), atrial fibrillation (AF), chronic heart failure (HF), history of stroke or myocardial infarction (MI) was assessed. Chronic kidney disease (CKD) was defined as a glomerular filtration rate (GFR) lower than 60 mL/min/1.73 m^2^ according to the Berlin Initiative Study 1 (BIS1) formula (13). Documented dementia was determined from routine clinical documentation, including pre-existing diagnoses in electronic medical records and ICD-10 codes assigned during hospitalization. This variable did not represent a study-specific dementia diagnosis. In patients evaluated during hospitalization, dementia diagnosis was based on clinical assessment performed by the geriatric team, cognitive screening results including MMSE and CDT, medical history, clinical symptoms, and exclusion of other potential causes of cognitive impairment when clinically indicated.

Comorbidities were estimated from electronic health records based on ICD-10 codes assigned during hospitalization. Vitamin D supplementation was self-reported at admission; information on dose, formulation, duration of supplementation and adherence was not available.

### 2.3 Measurements

The BMI was calculated by dividing the weight (in kilograms) by the height squared (in meters). The laboratory parameters were obtained upon patients’ admission. Serum 25(OH)D concentration was measured using the electrochemiluminescence immunoassay (Roche Diagnostics, Mannheim, Germany) on a Cobas e411 analyzer. According to the Endocrine Society guidelines (2011) (14), vitamin D status was classified as follows: deficiency < 20 ng/mL, insufficiency 20–29 ng/mL, sufficiency 30–50 ng/mL, and potential excess >50 ng/mL. Serum 25(OH)D was analyzed both as a continuous variable and in predefined categories for descriptive and nonparametric analyses. The intra-assay coefficient of variation (CV%) was <6%, and the inter-assay CV was <8%, according to the manufacturer’s data. The assay was calibrated against the NIST SRM 972 reference material. Morphology with white blood cells (WBC) count (with normal range 4–11˟10^9^/L) and hemoglobin (Hb) level (normal range 12–16 g/dL) was measured using the Sysmex XN 2000 analyzer (Kobe, Japan). Additionally, C-reactive protein (CRP) (normal range 0–5.0 mg/L), creatinine and albumin (normal level 35–52 g/L) concentration were determined using the Beckman Coulter Dx700 AU analyzer (Brea, CA, USA). GFR was calculated by the BIS1 formula.

Nutritional Risk Scale-2002 (NRS) was performed for every patient at admission (15). Cognitive performance was assessed using the Polish adaptation of MMSE (16) and CDT scored by the Sunderland 10-point system (17). All instruments were administered by the same psychologist trained in geriatric psychometrics. Tests were performed between 9 a.m. and 2 p.m. to minimize fatigue. The MMSE was initially scored using the standard raw total, ranging from 0 to 30 points, with lower scores indicating greater cognitive impairment. At the time of clinical assessment, the raw MMSE score was adjusted for age and years of education according to the correction proposed by Mungas et al. (18): adjusted MMSE score = raw MMSE score − 0.471 × (years of education − 12) + 0.131 × (age − 70). The resulting age- and education-adjusted MMSE score, rather than the unadjusted raw score, was entered into the research database and used in all statistical analyses. Throughout the manuscript, the outcome MMSE <24 refers to an age- and education-adjusted MMSE score below 24 points. Age was recorded as a separate variable in the research database and was included as a covariate in the multivariable models. Years of education were used to calculate the adjusted MMSE score during clinical assessment but were not retained as a separate standardized variable in the research database. The unadjusted raw MMSE score was likewise not retained. Consequently, educational attainment could not be examined independently or included as an additional covariate in the multivariable models. CDT scores range from 0 to 10, with higher scores reflecting better visuospatial and executive function. According to the literature, a threshold of <6 points was used to define impaired CDT performance (17, 19). Depressive symptoms were measured using the 15-item Geriatric Depression Scale (GDS) (20). Scores ≥6 were interpreted as indicative of clinically relevant depressive mood.

### 2.4 Statistical analysis

The normality of distribution was assessed using the Shapiro–Wilk test. As most continuous variables were not normally distributed, data are presented as mean ± standard deviation and median (interquartile range, IQR). Categorical variables were reported as absolute numbers and percentages.

Between-group comparisons were performed using two-sided Mann–Whitney *U* tests with continuity correction for continuous variables and two-sided Pearson’s chi-square tests for categorical variables. These preliminary bivariate analyses were used to identify variables associated with MMSE <24, CDT < 6, and documented dementia. Comparisons of cognitive test scores across categories of serum 25(OH)D concentration were conducted using the Kruskal–Wallis test, followed by post-hoc pairwise comparisons when appropriate. Associations between serum 25(OH)D concentrations and continuous clinical and laboratory variables were examined using Spearman’s rank correlation coefficients (rho), with analyses performed separately in women and men. As an additional exploratory analysis addressing seasonal variation, serum 25(OH)D concentrations were compared across months of blood sampling using the Kruskal–Wallis test. Final multivariable logistic regression models were used to examine factors associated with MMSE <24 points, CDT < 6 points, and documented dementia. The final covariate set included age, sex, BMI, serum albumin concentration, serum 25(OH)D concentration, GDS score, and history of stroke. To explore whether the association between serum 25(OH)D concentration and cognitive outcomes was compatible with a threshold-related pattern, additional category-based multivariable logistic regression models were performed. Serum 25(OH)D concentration was categorized as <10 ng/mL, 10–19 ng/mL, 20–29 ng/mL, and ≥30 ng/mL. The category ≥30 ng/mL was used as the reference group. These models were adjusted for the same covariates as the primary models, except that serum 25(OH)D concentration was entered as a categorical rather than continuous variable. Collinearity was assessed using variance inflation factors (VIFs). Model performance and fit were evaluated using likelihood-ratio model *χ*^2^ statistics, the Hosmer–Lemeshow goodness-of-fit test, Pearson *χ*^2^/df, Nagelkerke *R*^2^, and receiver operating characteristic curve analysis with calculation of the area under the curve (AUC).

Sensitivity analyses were conducted by excluding participants with a history of stroke, by excluding participants receiving vitamin D supplementation, and by restricting the analysis to participants with normal CRP concentration (≤5 mg/L; *n* = 448). To further explore the potential role of inflammation, additional exploratory multivariable logistic regression models were performed including elevated CRP status and the interaction term between serum 25(OH)D concentration and elevated CRP status. Elevated CRP was defined as CRP > 5 mg/L. The interaction term was calculated as serum 25(OH)D concentration × elevated CRP status. These models included the same covariates as the primary models. To formally assess whether sex modified the associations between serum 25(OH)D concentration and cognitive outcomes, additional exploratory multivariable logistic regression models were fitted separately for adjusted MMSE <24, CDT < 6, and documented dementia. Each model included serum 25(OH)D concentration, sex, and the serum 25(OH)D × sex interaction term and was adjusted for the same covariates as the corresponding primary model. The interaction term was calculated as serum 25(OH)D concentration, per 1 ng/mL, multiplied by male sex (male = 1, female = 0).

To address the possibility of residual confounding by broader comorbidity burden, additional exploratory extended multivariable models were also performed. These models included the primary covariates and additional recorded vascular, renal, nutritional, and inflammatory variables: NRS score, elevated CRP status >5 mg/L, arterial hypertension, diabetes mellitus, coronary artery disease, atrial fibrillation, heart failure, chronic kidney disease, and myocardial infarction. These extended models were interpreted as exploratory because of potential multicollinearity and overadjustment in a hospitalized geriatric population with a high burden of interrelated comorbidities.

The same multivariable logistic regression models as in the primary analysis were used in all sensitivity analyses. Statistical significance was defined as a two-sided *p*-value ≤ 0.05. All analyses were performed using Statistica 13.3 (StatSoft, Kraków, Poland).

### 2.5 Ethical consideration

The study was conducted according to the guidelines of the Declaration of Helsinki and approved by the Ethics Committee of the Medical University of Lodz (approval number: RNN/68/23/KE, 18 April 2023).

## 3 Results

### 3.1 Baseline characteristics of the study population

Table 1 presents the general characteristics of the study group according to sex. Women were significantly older and had lower hemoglobin concentration, worse kidney function assessed by GFR, and higher GDS scores compared to men. In contrast, men had lower serum 25(OH)D concentrations and higher CRP levels. In cognitive tests, a significant difference was found in CDT results (better in men), while MMSE scores did not differ significantly. Regarding comorbidities, arterial hypertension, HF and CKD were more prevalent among women, whereas DM and history of myocardial infarction were more common in men. Additionally, women significantly more often used vitamin D supplementation. Overall, 25(OH)D deficiency (<20 ng/mL) was present in 53.3% of participants, and only 27.6% had concentrations ≥30 ng/mL.

**Table 1:** General characteristics of study group according to sex (*N* = 1,539).

Variable	Women (*n* = 1,057)	Men (*n* = 482)	*p*-value
	Mean ± SD, median (lower; upper quartiles)	Mean ± SD, median (lower; upper quartiles)	
Age, years	82.89 ± 8.07	79.12 ± 8.12	<0.001^a^
	84 (77; 89)	78 (73; 86)	
Serum 25(OH)D, ng/mL	24.05 ± 17.45	18.37 ± 14.89	<0.001^a^
	20.9 (10.5; 33.3)	14.25 (8.07; 24.09)	
BMI, kg/m^2^	25.90 ± 5.75	25.65 ± 5.16	0.91^a^
	25.11 (22.22; 29.09)	24.88 (22.60; 28.03)	
Hb, g/dL	11.49 ± 1.97	11.73 ± 2.47	0.016^a^
	11.6 (10.2; 12.9)	11.8 (10.1; 13.5)	
GFR, mL/min/1.73 m^2^	52.22 ± 25.70	57.33 ± 25.01	<0.001^a^
	48.2 (37.8; 62.8)	54.0 (39.1; 69.9)	
CRP, mg/L	44.81 ± 65.36	60.93 ± 69.25	<0.001^a^
	13.7 (3.1; 65.4)	28.5 (6.3; 97.4)	
Albumin, g/L	34.20 ± 6.03	33.60 ± 6.29	0.11^a^
	34.4 (30.1; 38.5)	33.7 (29.9; 38.0)	
GDS, points	5.94 ± 3.8	5.31 ± 3.73	0.005^a^
	5.0 (3.0; 9.0)	4.0 (2.0–8.0)	
Documented dementia, number (%)	557 (52.7%)	226 (46.9%)	0.03^b^
Arterial hypertension, number (%)	866 (81.93%)	337 (69.92%)	<0.001^b^
Lipid disorders, number (%)	371 (35.1%)	150 (31.12%)	0.12^b^
Diabetes mellitus, number (%)	325 (30.75%)	173 (35.89%)	0.045^b^
Coronary artery disease, number (%)	284 (26.87%)	147 (30.50%)	0.14^b^
Myocardial infarction, number (%)	80 (7.57%)	87 (18.05%)	<0.001^b^
Atrial fibrillation, number (%)	340 (32.17%)	137 (28.42%)	0.14^b^
Heart failure, number (%)	780 (73.79%)	332 (68.88%)	0.045^b^
Stroke, number (%)	192 (18.16%)	87 (18.05%)	0.96^b^
Chronic kidney disease, number (%)	725 (68.59%)	299 (62.03%)	0.01^b^
MMSE, points	19.58 ± 8.24	20.29 ± 8.27	0.08^a^
	22.0 (15.0; 26.0)	23.0 (16.0; 26.0)	
CDT, points	4.42 ± 3.88	5.10 ± 3.99	0.006^a^
	4.0 (0; 8.0)	5.0 (0; 10.0)	
Self-reported vitamin D supplementation, number (%)	155 (14.66%)	45 (9.37%)	0.003^b^

MMSE—Mini-Mental State Examination; CDT—Clock-Drawing Test; SD—standard deviation.

a Mann–Whitney *U* test.

b Chi-square test.

MMSE values refer to age- and education-adjusted scores calculated using the Mungas correction.

### 3.2 Serum 25(OH)D concentration according to cognitive outcomes, selected clinical conditions, and month of blood sampling

MMSE < 24 was observed in 50.9%, CDT < 6 in 47.6%, and documented dementia was found in 50.9% of participants. Serum 25(OH)D concentrations were significantly lower in patients with cognitive impairment (MMSE < 24), documented dementia, and elevated CRP levels (all *p* ≤ 0.002), both in women and men. For CDT < 6 points, lower vitamin D levels were observed in women and in the overall group, but not in men. Men with a history of stroke had significantly lower serum 25(OH)D concentration, while in women and in total sample, serum 25(OH)D concentrations did not differ significantly between patients with and without prior stroke (Table 2).

**Table 2:** Serum 25(OH)D concentration (ng/mL) in patients with selected clinical conditions.

Variable	Sex	Present condition	Absent condition	*p*-value
		Mean ± SD, median (lower; upper quartiles)	Mean ± SD, median (lower; upper quartiles)	
MMSE < 24 points	Women	22.44 ± 16.09	27.55 ± 18.92	<0.001^a^
		19.5 (9.0–32.0)	24.25 (14.45–35.4)	
	Men	17.30 ± 15.32	20.16 ± 14.47	0.006^a^
		12.9 (6.9–24.3)	16.9 (10.4–25.9)	
	Whole group	20.96 ± 16.03	25.18 ± 17.94	<0.001^a^
		17.5 (8.32–30.3)	21.4 (12.6–33.3)	
CDT < 6 points	Women	23.34 ± 15.81	27.77 ± 19.67	<0.001^a^
		20.7 (10.1–32.8)	24.6 (15.2–35.45)	
	Men	18.79 ± 16.81	19.48 ± 13.37	0.15^a^
		14.0 (7.2–26.0)	16.4 (9.97–25.7)	
	Whole group	22.08 ± 16.21	24.80 ± 18.10	0.002^a^
		18.6 (9.5–31.4)	21.2 (12.3–32.8)	
Documented dementia	Women	22.14 ± 15.92	26.18 ± 18.80	<0.001^a^
		19.0 (8.8–31.9)	22.5 (12.7–34.5)	
	Men	16.38 ± 12.92	20.12 ± 16.26	0.002^a^
		12.55 (6.8–22.6)	16.4 (9.5–27.4)	
	Whole group	20.48 ± 15.33	24.13 ± 18.20	<0.001^a^
		16.9 (8.2–29.9)	20.5 (11.1–32.8)	
History of stroke	Women	24.81 ± 17.04	23.88 ± 17.55	0.39^a^
		22.4 (10.5–36.2)	20.8 (10.5–32.9)	
	Men	15.00 ± 10.33	19.11 ± 15.63	0.03^a^
		12.6 (6.3–20.7)	14.7 (8.7–25.1)	
	Whole group	21.75 ± 15.91	22.39 ± 17.11	0.63^a^
		18.1 (8.4–32.0)	18.6 (9.8–31.3)	
CRP > 5 mg/L	Women	21.72 ± 15.68	28.60 ± 19.49	<0.001^a^
		19.0 (9.0–31.6)	26.3 (14.8–37.9)	
	Men	17.28 ± 14.96	22.17 ± 14.27	<0.001^a^
		13.2 (6.9–23.5)	18.6 (12.4–29.3)	
	Whole group	20.20 ± 15.57	27.04 ± 18.55	<0.001^a^
		16.9 (8.1–29.2)	23.4 (13.7–34.9)	
Vitamin D supplementation	Women	33.35 ± 22.09	22.4 ± 16.01	<0.001^a^
		31.6 (19.6–44.8)	19.3 (9.8–31.8)	
	Men	25.65 ± 18.54	17.61 ± 14.29	0.002^a^
		20.5 (10.9–34.2)	13.9 (7.6–24.0)	
	Whole group	31.61 ± 21.54	20.84 ± 15.63	<0.001^a^
		29.6 (21.5–42.7)	17.5 (9.0–29.4)	

MMSE—Mini-Mental State Examination; CDT—Clock-Drawing Test; SD—standard deviation.

a Mann–Whitney *U* test.

MMSE <24 was defined using the age- and education-adjusted MMSE score.

In an additional exploratory analysis addressing seasonal variation, serum 25(OH)D concentrations were compared across months of blood sampling. No statistically significant month-to-month differences were observed [Kruskal–Wallis *H* (11, *N* = 1,539) = 17.32, *p* = 0.099].

### 3.3 Distribution of 25(OH)D categories according to cognitive test results

**Figure 2:**
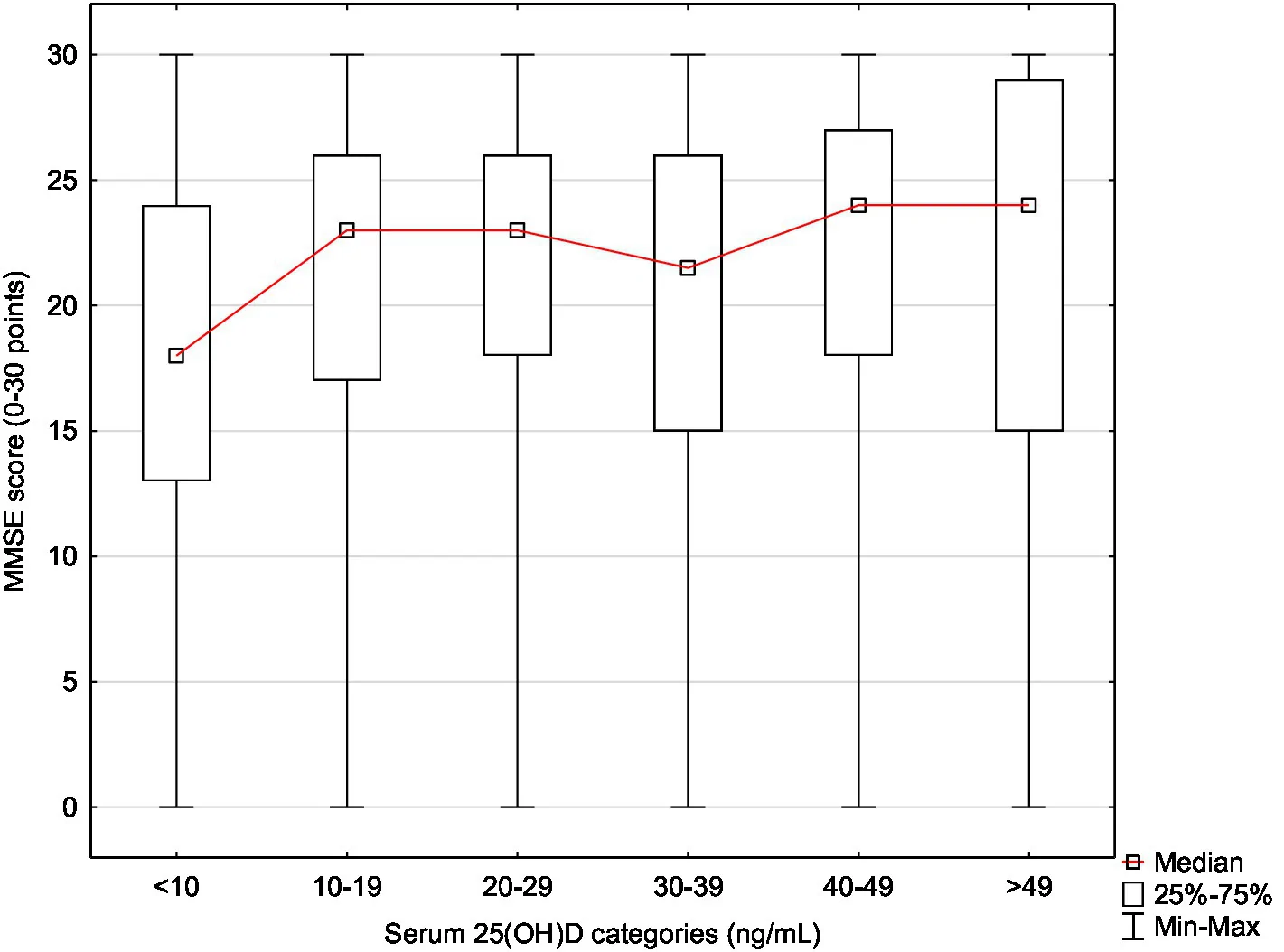
Distribution of Mini-Mental State Examination (MMSE) scores across categories of serum 25-hydroxyvitamin D [25(OH)D] concentration.

Participants were distributed across 25(OH)D categories as follows: 408 (26.5%) had concentrations <10 ng/mL, 412 (26.8%) 10–19 ng/mL, 295 (19.2%) 20–29 ng/mL, 217 (14.1%) 30–39 ng/mL, 126 (8.2%) 40–49 ng/mL, and 81 (5.3%) ≥ 50 ng/mL. Both MMSE and CDT scores increased across increasing categories of serum 25(OH)D, with the lowest scores observed in participants with severe deficiency. Cognitive performance differed significantly across categories of serum 25(OH)D concentration. For MMSE, the Kruskal–Wallis test showed a significant overall difference between vitamin D categories (*H* = 51.26, *p* < 0.001), with progressively higher ranks across increasing 25(OH)D categories (Figure 2).

**Figure 3:**
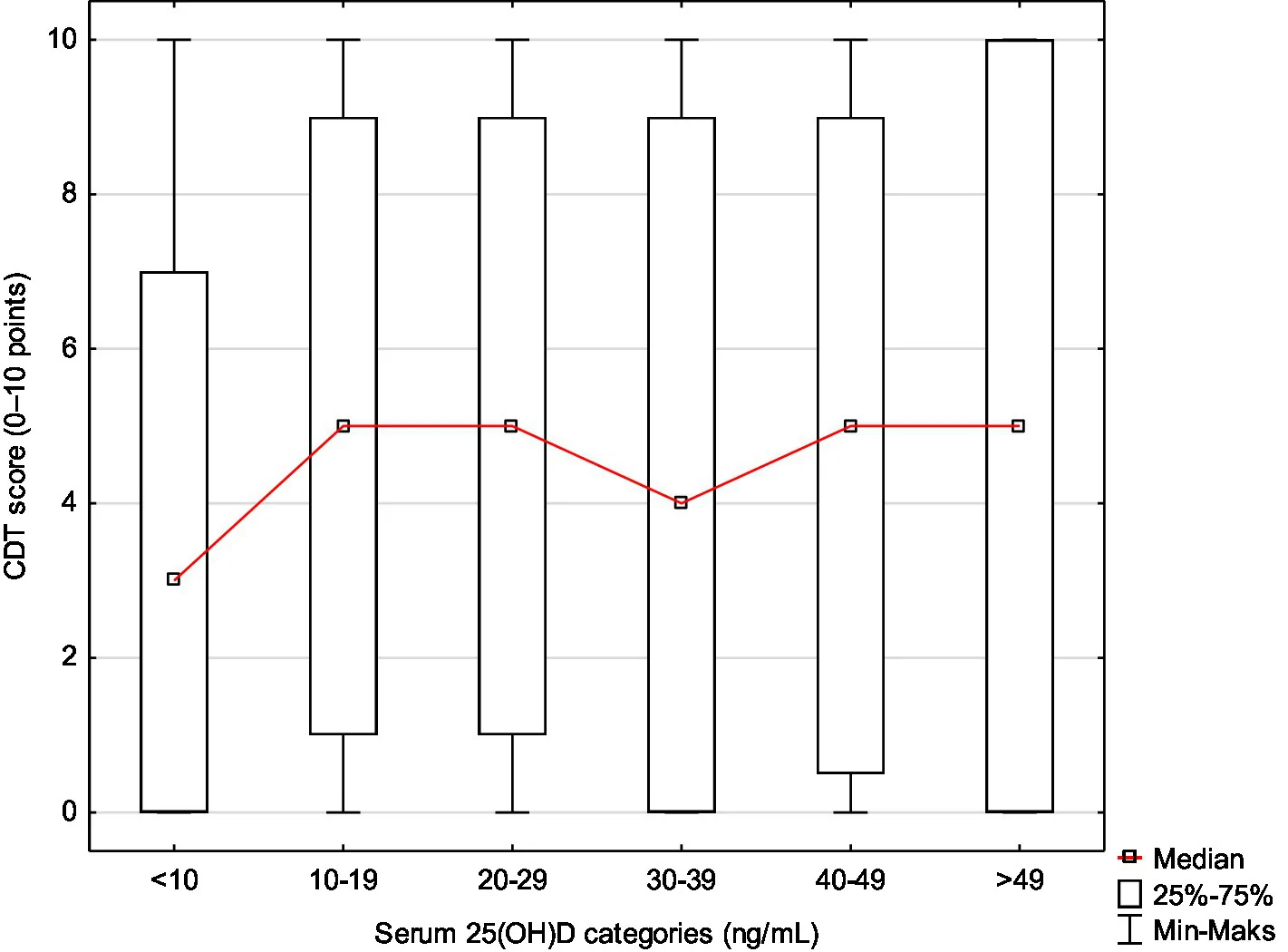
Distribution of Clock Drawing Test (CDT) scores across categories of serum 25-hydroxyvitamin D [25(OH)D] concentration.

Similarly, CDT scores differed significantly across 25(OH)D categories (Kruskal–Wallis *H* = 22.55, *p* = 0.0004), showing a parallel but less pronounced trend (Figure 3).

### 3.4 Associations between 25(OH)D concentration and continuous clinical and laboratory variables

Serum 25(OH)D concentrations showed significant correlations with several clinical parameters. In the whole group, positive correlations were observed with hemoglobin (*ρ* = 0.07, *p* = 0.005), albumin (*ρ* = 0.20, *p* < 0.001), MMSE (*ρ* = 0.11, *p* < 0.001), and CDT (ρ = 0.08, *p* = 0.008), while negative correlations were found with GFR (*ρ* = −0.06, *p* = 0.014) and CRP (*ρ* = −0.16, *p* < 0.001).

In sex-stratified analyses, these associations were more pronounced in women, particularly for hemoglobin, albumin, MMSE, CDT, and CRP (all *p* ≤ 0.01). In men, significant correlations were limited to GFR (*ρ* = −0.12, *p* = 0.01), CRP (*ρ* = −0.17, *p* = 0.001), and albumin (*ρ* = 0.16, *p* = 0.001). No significant associations were observed for GDS or BMI in any group.

### 3.5 Bivariate analyses for adverse cognitive outcomes

Preliminary two-sided bivariate analyses were performed to identify variables associated with adverse cognitive outcomes, including MMSE <24, CDT < 6, and documented dementia. In the whole study group, all three outcomes were significantly associated with age, BMI, serum 25(OH)D concentration, albumin concentration, and GDS score, as assessed using the Mann–Whitney U test with continuity correction. In general, adverse cognitive outcomes were associated with older age, lower BMI, lower serum 25(OH)D concentration, lower albumin concentration, and higher depressive symptom burden. Prior stroke was significantly associated with all three outcomes in chi-square analyses, whereas sex was significantly associated with CDT < 6 and documented dementia, but not with MMSE <24. In sex-stratified analyses, age, serum 25(OH)D concentration, albumin concentration, BMI, and GDS score were significantly associated with MMSE <24 in both women and men. For CDT < 6, age, albumin concentration, and GDS score remained significant in both sexes, whereas BMI and serum 25(OH)D concentration were significant only in women. For documented dementia, age, serum 25(OH)D concentration, and GDS score were significant in both sexes, while BMI and albumin concentration were significant only in women. Based on these analyses and clinical relevance, age, BMI, serum 25(OH)D concentration, albumin concentration, GDS score, prior stroke, and sex were considered candidate predictors for the multivariable logistic regression models.

### 3.6 Multivariable logistic regression models for cognitive impairment and documented dementia

Variables associated with cognitive outcomes in bivariate analyses were considered for inclusion in multivariable logistic regression models. Final models included age, BMI, serum albumin, serum 25(OH)D concentration, GDS score, sex, and prior stroke (Tables 3–5).

**Table 3:** Multivariable logistic regression model for odds of MMSE <24 points.

Predictor	OR (95% CI)	*p*-value
Age, per 1 year	1.080 (1.059–1.103)	<0.001
BMI, per 1 kg/m^2^	0.966 (0.938–0.995)	0.020
Serum 25(OH)D, per 1 ng/mL	0.984 (0.973–0.994)	0.001
Albumin, per 1 g/L	0.950 (0.923–0.978)	<0.001
GDS score, per 1 point	1.125 (1.080–1.171)	<0.001
Prior stroke, yes vs. no	1.525 (1.004–2.317)	0.048
Female sex, yes vs. male	1.056 (0.756–1.476)	0.748
Model *χ*^2^(7) = 133.81; *p* < 0.001.		

MMSE <24 was defined using the age- and education-adjusted MMSE score.

**Table 4:** Multivariable logistic regression model for CDT score <6.

Predictor	OR (95% CI)	*p*-value
Age, per 1 year	1.067 (1.045–1.089)	<0.001
BMI, per 1 kg/m^2^	0.980 (0.951–1.009)	0.170
Serum 25(OH)D, per 1 ng/mL	0.988 (0.978–0.998)	0.018
Albumin, per 1 g/L	0.966 (0.938–0.995)	0.021
GDS score, per 1 point	1.137 (1.090–1.187)	<0.001
Prior stroke, yes vs. no	1.711 (1.105–2.650)	0.016
Female sex, yes vs. male	1.314 (0.935–1.846)	0.116
Model *χ*^2^(7) = 106.21; *p* < 0.001.		

**Table 5:** Multivariable logistic regression model for odds of documented dementia.

Predictor	OR (95% CI)	*p*-value
Age, per 1 year	1.078 (1.056–1.100)	<0.001
BMI, per 1 kg/m^2^	0.963 (0.935–0.992)	0.012
Serum 25(OH)D, per 1 ng/mL	0.980 (0.970–0.990)	<0.001
Albumin, per 1 g/L	0.970 (0.943–0.997)	0.032
GDS score, per 1 point	1.079 (1.037–1.122)	<0.001
Prior stroke, yes vs. no	1.808 (1.201–2.724)	0.005
Female sex, yes vs. male	1.103 (0.791–1.539)	0.562
Model *χ*^2^(7) = 137.53; *p* < 0.001.		

In multivariable logistic regression analysis, serum 25(OH)D concentration was associated with lower odds of MMSE score <24 after adjustment for age, sex, albumin concentration, depressive symptoms, BMI, and prior stroke (OR = 0.984, 95% CI: 0.973–0.994, *p* = 0.001). This corresponds to 15% lower odds of having MMSE score <24 points with each 10 ng/mL increase in serum 25(OH)D concentration. Older age, higher GDS scores and history of stroke were associated with worse MMSE performance, whereas higher albumin concentration and BMI were associated with better cognitive performance. The overall multivariable model was statistically significant [*χ*^2^(7) = 133.81, *p* < 0.001] (Table 3).

In the adjusted model for CDT < 6 points, higher serum 25(OH)D concentration was associated with lower odds of poor CDT performance after adjustment for covariates (OR = 0.988 per 1 ng/mL increase; 95% CI: 0.978–0.998; *p* = 0.018). This translates to 11% reduction in the odds of poor CDT performance (<6 points) for each 10 ng/mL increase in 25(OH)D concentration. Older age, higher GDS scores, and a history of stroke were associated with worse CDT results, whereas higher albumin concentration was related to better cognitive outcomes. BMI was not a significant predictor in this model. The overall multivariable model was statistically significant [*χ*^2^(7) = 106.21; *p* < 0.001] (Table 4).

In the multivariable model for documented dementia, higher serum 25(OH)D concentration was associated with lower odds of documented dementia after adjustment for selected covariates (OR = 0.980 per 1 ng/mL increase; 95% CI: 0.970–0.990; *p* < 0.001). This corresponds to approximately an 18% reduction in the odds of documented dementia for each 10 ng/mL increase in 25(OH)D concentration. Older age, higher GDS scores, and prior stroke were associated with increased odds of documented dementia, whereas higher albumin concentration and BMI were associated with decreased odds. The overall model was statistically significant [*χ*^2^(7) = 137.53; *p* < 0.001] (Table 5). Diagnostic measures for the primary multivariable logistic regression models are presented in Supplementary Table S2.

### 3.7 Category-based multivariable logistic regression models according to serum 25(OH)D categories

In additional category-based multivariable logistic regression models, serum 25(OH)D concentration was analyzed using clinically interpretable categories, with ≥30 ng/mL used as the reference group. Severe 25(OH)D deficiency (<10 ng/mL) was associated with higher odds of MMSE <24 compared with serum 25(OH)D ≥ 30 ng/mL (OR = 1.800, 95% CI: 1.131–2.866, *p* = 0.017). The categories 10–19 ng/mL and 20–29 ng/mL were not significantly associated with MMSE <24.

In the corresponding model for CDT < 6, none of the serum 25(OH)D categories was significantly associated with impaired CDT performance compared with the reference category. For documented dementia, severe 25(OH)D deficiency (<10 ng/mL) was associated with higher odds of documented dementia (OR = 2.637, 95% CI: 1.680–4.140, *p* < 0.001), whereas the categories 10–19 ng/mL and 20–29 ng/mL were not statistically significant. These findings suggest that the association may be more pronounced in participants with severe 25(OH)D deficiency rather than following a strictly linear pattern across all categories. Detailed results are presented in Supplementary Table S3.

### 3.8 Sensitivity analyses

#### 3.8.1 Exclusion of patients with a history of stroke

After excluding participants with a history of stroke, serum 25(OH)D concentration remained associated with lower odds of MMSE <24 (OR = 0.983 per 1 ng/mL increase; 95% CI: 0.973–0.994; *p* = 0.003). The association also remained significant for CDT < 6 (OR = 0.989; 95% CI: 0.978–0.999; *p* = 0.034) and documented dementia (OR = 0.978; 95% CI: 0.967–0.990; *p* < 0.001). These findings indicate that the observed associations between serum 25(OH)D concentration and adverse cognitive outcomes were not driven solely by participants with prior stroke (Supplementary Table S4).

#### 3.8.2 Exclusion of participants receiving vitamin D supplementation

In sensitivity analyses restricted to participants not receiving vitamin D3 supplementation, the main findings remained materially unchanged. Higher serum 25(OH)D concentration continued to be associated with lower odds of MMSE <24 (OR = 0.985; 95% CI: 0.974–0.996; *p* = 0.009), CDT < 6 (OR = 0.989; 95% CI: 0.977–1.000; *p* = 0.048), and documented dementia (OR = 0.981; 95% CI: 0.970–0.993; *p* = 0.001).

Age and depressive symptoms (GDS score) remained strong and consistent predictors of adverse cognitive outcomes across all models. Higher BMI was associated with lower odds of MMSE <24 and documented dementia. Higher albumin concentration was significantly associated with lower odds of MMSE <24, whereas its associations with CDT < 6 and documented dementia were not statistically significant. Prior stroke remained significantly associated with higher odds of CDT < 6 and documented dementia, but not with MMSE <24 in this subgroup. Sex was not significantly associated with any of the cognitive outcomes (Supplementary Table S5).

#### 3.8.3 Restriction to participants with normal CRP concentration

In sensitivity analyses restricted to participants with normal CRP concentration (≤5 mg/L; *n* = 448, including 339 women and 109 men), the associations between serum 25(OH)D concentration and adverse cognitive outcomes were attenuated. Higher serum 25(OH)D concentration showed a consistent direction of association with lower odds of MMSE <24, CDT < 6, and documented dementia; however, these associations did not reach statistical significance in this subgroup. Age and GDS score remained significantly associated with adverse cognitive outcomes, while BMI and albumin concentration were inversely associated with selected outcomes. Detailed results are presented in Supplementary Table S6.

#### 3.8.4 Exploratory interaction analysis between serum 25(OH)D concentration and elevated CRP status

In additional exploratory models including elevated CRP status and the interaction term between serum 25(OH)D concentration and elevated CRP status, no statistically significant interactions were observed for MMSE < 24 (OR = 0.990, 95% CI: 0.972–1.009, *p* = 0.317) or CDT < 6 (OR = 0.998, 95% CI: 0.979–1.018, *p* = 0.868). For documented dementia, the interaction term approached but did not reach statistical significance (OR = 0.980, 95% CI: 0.960–1.000, *p* = 0.054). These results do not provide formal evidence of significant effect modification by elevated CRP status. Detailed results are presented in Supplementary Table S7.

#### 3.8.5 Extended multivariable models including additional comorbidity, nutritional, and inflammatory variables

In additional exploratory extended multivariable models, we further adjusted for major vascular, renal, nutritional, and inflammatory variables, including NRS score, elevated CRP status, arterial hypertension, diabetes mellitus, coronary artery disease, atrial fibrillation, heart failure, chronic kidney disease, and myocardial infarction. In these extended models, serum 25(OH)D concentration remained significantly associated with MMSE < 24 (OR = 0.988, 95% CI: 0.979–0.998, *p* = 0.021) and documented dementia (OR = 0.978, 95% CI: 0.968–0.989, *p* < 0.001). The association with CDT < 6 was attenuated and no longer statistically significant (OR = 0.993, 95% CI: 0.983–1.003, *p* = 0.171). These findings suggest that the associations with global cognitive impairment and documented dementia were not fully explained by the additional comorbidity, nutritional, and inflammatory variables included in the extended models, whereas the CDT-related association was less robust. Detailed results are presented in Supplementary Table S8.

#### 3.8.6 Exploratory interaction analyses between serum 25(OH)D concentration and sex

Formal interaction analyses provided no evidence that sex modified the associations between serum 25(OH)D concentration and adjusted MMSE <24 (OR for interaction = 1.000, 95% CI: 0.980–1.022; p for interaction = 0.966), CDT < 6 (OR = 0.988, 95% CI: 0.968–1.008; *p* = 0.239), or documented dementia (OR = 1.024, 95% CI: 0.997–1.052; *p* = 0.081). Detailed results are presented in Supplementary Table S9.

## 4 Discussion

To our knowledge, this is one of the few studies in a hospitalized geriatric population showing a graded cross-sectional association between serum 25(OH)D categories and cognitive performance. In this large hospital-based geriatric cohort, we found that lower serum 25(OH)D concentrations were associated with worse global cognitive performance, poorer executive/visuospatial function, and higher odds of documented dementia after adjustment for selected covariates. Additional sensitivity analyses addressed the potential influence of prior stroke, vitamin D supplementation, and systemic inflammation.

In the general characteristics of our study sample, a sex difference was observed in CDT performance, with men scoring higher than women; however, MMSE scores did not differ significantly between sexes. This pattern may be explained by the fact that the men in our cohort were significantly younger, and, as reported in the literature, men tend to perform better on visuospatial cognitive tasks such as the CDT (21). Moreover, women in our sample had significantly higher scores on the GDS, which is consistent with epidemiological findings (22). Elevated depressive symptoms in women may contribute to poorer cognitive test performance or coexist with cognitive impairment (23).

Regarding serum 25(OH)D concentrations, women had significantly higher serum vitamin D concentrations than men, despite being older. Several studies have reported similar findings (24, 25). However, research on the German population (26) has found no significant sex differences in serum 25(OH)D concentrations, indicating that such differences may depend on population characteristics, lifestyle factors, and supplementation practices. In our cohort, women more frequently reported vitamin D supplementation, which is in line with data from the literature (27) and may be one of the likely explanations.

In bivariate analysis, significantly lower 25(OH)D concentration in patients with MMSE <24 points and participants with documented dementia in both sexes is consistent with meta-analytic and observational findings that lower vitamin D status is associated with poorer cognitive performance or increased risk of cognitive decline and dementia (9, 28). Llewellyn et al. (29) showed that low levels of 25(OH)D were associated with substantial cognitive decline in the older population studied over 6 years. Significantly lower 25(OH)D levels in patients with CDT < 6 scores were observed in the whole group and in women, whereas this difference did not reach statistical significance in men. The observed sex differences may be partially explained by larger representation of women in the study population. On the other hand, many studies assessing the effects of vitamin D on clinical conditions have demonstrated different outcomes in men and women (24). However, despite the sex-related differences observed in some bivariate analyses, formal interaction tests did not demonstrate statistically significant effect modification by sex for any of the three cognitive outcomes. Patients with increased CRP had significantly lower 25(OH)D concentration. A literature review supports our findings – in a large cohort of older adults (ELSA study), there was a significant inverse association between 25(OH)D and CRP (30). Inflammation could shift the balance toward enhanced degradation of vitamin D metabolites, lowering circulating 25(OH)D. Our observation that men after stroke had lower 25(OH)D levels aligns with broader epidemiological and biological data (24, 31).

The observed monotonic increase in both MMSE and CDT scores across ascending categories of serum 25(OH)D concentration suggests a monotonic association between vitamin D status and cognitive performance. Participants with severe 25(OH)D deficiency exhibited the poorest cognitive outcomes, whereas progressively higher vitamin D categories were associated with better global cognition and visuospatial/executive functioning. This gradient pattern strengthens the biological plausibility of the association and is consistent with previous observational studies demonstrating lower cognitive scores and higher prevalence of cognitive impairment among individuals with low 25(OH)D levels (32). The trend observed for CDT may reflect differences in sensitivity between global cognitive screening (MMSE) and domain-specific visuospatial/executive assessments, as well as potential sex-related influences on CDT performance. Although causality cannot be inferred from this cross-sectional analysis, the consistency of the level–response pattern across two independent cognitive measures supports further evaluation of the relationship between 25(OH)D status and cognitive outcomes in older adults.

The category-based multivariable models further suggested that the association was most evident among participants with severe 25(OH)D deficiency. Compared with serum 25(OH)D ≥ 30 ng/mL, concentrations <10 ng/mL were associated with higher odds of MMSE < 24 and documented dementia, whereas intermediate categories were not consistently associated with adverse cognitive outcomes. This pattern suggests a possible threshold-related association, although it should be interpreted cautiously because of the cross-sectional design and residual confounding.

Several biological mechanisms may provide plausibility for an association between vitamin D status and cognitive outcomes, including vitamin D receptor expression in the brain, regulation of neuroinflammatory and oxidative pathways, neurotrophic signaling, and potential vascular effects (33, 34). However, these mechanisms were not directly assessed in the present study. Therefore, in this hospitalized geriatric population, serum 25(OH)D should be interpreted not only as a potential biological correlate of cognitive outcomes, but also as a marker of nutritional status, inflammatory burden, frailty, multimorbidity, and overall health status (33, 34).

In correlation analysis in both sexes, positive association was observed between serum 25(OH)D and hemoglobin and albumin levels, suggesting that higher vitamin D concentration may reflect better overall nutritional and hematological status, as albumin is both a marker of nutrition and a major carrier of vitamin D in circulation. Sanson et al. (35) found that lower albumin levels are strongly associated with lower 25(OH)D and worse outcomes in older COVID-19 patients. Another publication (36) demonstrates that vitamin D insufficiency was associated with significantly higher odds of anemia (OR = 2.4). Albumin concentration was associated with cognitive outcomes in the primary multivariable models, corroborating evidence that nutritional status is related to cognitive aging (37). Negative relationships were found between vitamin D and CRP levels, supporting our findings from bivariate analysis. Overall, vitamin D levels were modestly associated with markers of nutritional status, inflammation, and cognitive performance, with stronger and more consistent relationships in women likely reflecting their larger representation in the study population. Albumin should be interpreted as a broad marker of nutritional status, inflammation, frailty, and disease severity rather than as a specific mechanistic factor. Because albumin is also involved in the circulation of vitamin D metabolites, adjustment for albumin may reduce confounding by general health status, but it may also introduce partial overadjustment if albumin lies on pathways linking disease burden, vitamin D metabolism, and cognitive outcomes.

In the multivariable logistic model, older age, lower albumin and serum 25(OH)D concentrations, higher GDS scores, and prior stroke were associated with higher odds of MMSE <24 points and documented dementia, while sex was not significant in those models. In the model with CDT < 6 points as the dependent variable, the effect estimate for 25(OH)D was weaker, and BMI and sex were not significant. Age is a well-known risk factor of dementia (38, 39). It drives multiple converging biological processes that lower brain resilience and promotes neurodegeneration (40). Multiple epidemiological studies indicate that lower serum albumin levels correlate with worse cognitive performance and higher risk of dementia (41, 42). In a large population-based sample (England), individuals in the lowest albumin quartile had significantly higher odds of cognitive impairment compared with the highest quartile, independent of age and vascular risk factors (43). In older adults with newly diagnosed Alzheimer’s disease, lower albumin levels were associated with poorer cognitive test scores when controlling other factors (44). Albumin acts as an amyloid-β binding and clearance modulator. Low serum albumin reduces the capacity to bind amyloid-β, impairing its efflux from the brain, leading to higher cerebral Aβ deposition (45). Besides that, albumin serves as a major antioxidant in blood, scavenging free radicals (46). Numerous findings support our results that depression is not merely comorbid with dementia but is epidemiologically associated with increased risk (47). There may be common neurobiological processes (e.g., neurotransmitter changes, inflammation, and vascular pathology) that contribute to both depression and dementia (48). Multiple large studies and meta-analyses show that individuals with a history of stroke are significantly more likely to develop dementia (49–51). A stroke causes focal cerebral ischemia or hemorrhage, injuring neurons and glial cells in critical cognitive regions (e.g., hippocampus, frontal cortex). This reduces cognitive reserve, making clinical dementia more likely once neurodegenerative processes accumulate (52).

The persistence of the association between serum 25(OH)D concentration and cognitive outcomes after adjustment for selected covariates suggests that vitamin D status may be a clinical marker associated with cognitive vulnerability in hospitalized older adults. However, because of the cross-sectional design, reverse causation and residual confounding cannot be excluded. Lower 25(OH)D levels may also reflect reduced mobility, lower sunlight exposure, poorer nutritional intake, inflammation, or greater overall disease burden in patients with cognitive impairment. The magnitude of the observed associations should also be considered. Although higher serum 25(OH)D concentration was statistically significantly associated with lower odds of MMSE < 24, CDT < 6, and documented dementia in the primary models, the effect sizes were modest. The odds ratios per 1 ng/mL increase were close to 1.0, corresponding to approximately 11–18% lower odds of adverse cognitive outcomes per 10 ng/mL higher serum 25(OH)D concentration.

The sensitivity analysis excluding participants receiving vitamin D supplementation indicated that the observed associations between serum 25(OH)D concentration and cognitive outcomes were not driven by vitamin D supplementation. In sensitivity analyses excluding participants with a history of stroke, serum 25(OH)D concentration remained associated after adjustment for selected covariates with lower odds of MMSE < 24, CDT < 6, and documented dementia, supporting the robustness of the observed associations. The association with CDT was weaker than that observed for MMSE and documented dementia, with an effect size close to the threshold of statistical significance. This may reflect differences in measurement properties between global cognitive screening and domain-specific visuospatial and executive tests. CDT is characterized by greater measurement variability and may be influenced by visual, motor, and affective factors, which could reduce its sensitivity to vitamin D–related cognitive differences.

In the multivariable sensitivity analysis restricted to participants with normal CRP concentration, the associations between serum 25(OH)D and adverse cognitive outcomes were attenuated and did not reach statistical significance, although the direction of the effects remained consistent with the primary analyses. This attenuation may reflect reduced statistical power due to the smaller subgroup size, but it may also suggest that systemic inflammation partly contributes to or modifies the relationship between vitamin D status and cognitive outcomes (53). In additional exploratory models, we also tested the interaction between serum 25(OH)D concentration and elevated CRP status. The interaction terms were not statistically significant for MMSE < 24 or CDT < 6, while the interaction for documented dementia approached but did not reach statistical significance. These findings do not confirm formal effect modification by elevated CRP status. However, together with the attenuation observed in participants with normal CRP concentration, they suggest that systemic inflammation may contribute to the observed relationship between vitamin D status and cognitive outcomes. These findings suggest that systemic inflammation may be one factor contributing to the observed associations between serum 25(OH)D concentration and cognitive outcomes in hospitalized older adults.

The discovery that immune cells express both CYP27B1 and the vitamin D receptor (VDR) has established vitamin D as an important regulator of immune function (54). Macrophages, monocytes, dendritic cells, and lymphocytes are capable of locally converting circulating 25(OH)D into its active form, 1,25(OH)₂D, which subsequently binds to VDR and regulates the expression of genes involved in innate and adaptive immunity. Consequently, inadequate vitamin D status may impair immune defense (55) while promoting chronic low-grade inflammation, mechanisms that have been implicated in aging-related conditions including frailty and cognitive decline (56). Recent mechanistic literature also highlights the overlap between neurodegenerative, vascular, metabolic, and inflammatory pathways in cognitive impairment. Alzheimer’s disease and atherosclerosis may share mechanisms involving endothelial dysfunction, blood–brain barrier disruption, neurovascular inflammation, oxidative stress, autophagy dysregulation, and PI3K/AKT/mTOR-related signaling. Dysregulation of the PI3K/AKT/GSK3β axis has also been linked to brain insulin resistance, tau hyperphosphorylation, amyloid-β accumulation, and synaptic dysfunction, while gut–brain axis disturbances may contribute to cognitive impairment through systemic inflammation, gut dysbiosis, and neuroinflammation. Although these pathways were not directly assessed in our study, they provide a contemporary biological context for interpreting low serum 25(OH)D concentration as a marker of broader vascular, inflammatory, metabolic, and neurodegenerative vulnerability in hospitalized older adults (56–58).

## 5 Strengths and limitations

The main strengths of this study include the large sample size, consecutive recruitment of hospitalized older adults, standardized serum 25(OH)D measurement, use of validated cognitive screening tools, and several sensitivity and exploratory analyses addressing stroke history, vitamin D supplementation, systemic inflammation, nonlinear exposure patterns, model diagnostics, and missing-data-related selection bias. In contrast to many previous studies focused on community-dwelling or disease-specific populations, this study examined a broad geriatric hospital population with high multimorbidity and nutritional vulnerability, which increases its clinical relevance.

Several limitations should also be acknowledged. First, the cross-sectional observational design precludes causal inference, and reverse causation cannot be excluded. Lower serum 25(OH)D concentration may reflect reduced mobility, lower sunlight exposure, poorer nutritional intake, frailty, inflammation, or greater disease burden in patients with cognitive impairment or documented dementia. Residual confounding is also possible, as comorbidity burden, disease severity, therapeutic interventions, and frailty-related factors were not fully captured.

Second, the MMSE outcome was based on the age- and education-adjusted score calculated according to the Mungas correction. Although age was recorded separately and included in the multivariable models, years of education were not retained as a separate standardized variable in the research database. Therefore, educational attainment could not be examined independently or included as an additional covariate, and residual confounding related to education and cognitive reserve cannot be completely excluded. In addition, vitamin D supplementation was self-reported and data on dose, formulation, duration, and adherence were unavailable, which may have introduced exposure misclassification and residual confounding related to health-seeking behaviors.

Third, season or month of blood sampling was not included in the primary multivariable models. Although participants were recruited consecutively and exploratory analyses did not show significant month-to-month differences in serum 25(OH)D concentration, residual confounding related to sunlight exposure, outdoor activity, and seasonal variation cannot be excluded. Similarly, CRP was not included in the primary models because it may reflect acute illness and lie partly on the pathway between disease burden, vitamin D status, and cognitive performance. Therefore, inflammation was addressed in sensitivity analyses restricted to participants with normal CRP concentration and in exploratory interaction models including elevated CRP status.

Fourth, documented dementia was derived from routine clinical documentation and ICD-10 coding rather than from a standardized study-specific diagnostic assessment. Because MMSE and CDT findings may have contributed to the routine clinical diagnosis of dementia during hospitalization, partial incorporation bias is possible. Consequently, documented dementia was not fully independent of the cognitive test results, and the consistency of findings across these outcomes should not be interpreted as independent replication. Diagnostic heterogeneity and outcome misclassification cannot be excluded, and documented dementia should not be interpreted as incident or independently adjudicated dementia. The predominance of women and recruitment from a single geriatric department may also limit generalizability. Although formal interaction analyses did not demonstrate statistically significant effect modification by sex, the smaller number of men may have limited the precision and statistical power of these analyses.

Finally, albumin was included as a clinically relevant marker of nutritional and general health status, but it may also reflect inflammation, frailty, and disease severity; therefore, both residual confounding and partial overadjustment remain possible. Associations with CDT were less robust in sensitivity analyses and should be interpreted cautiously. Because multiple bivariate, subgroup, category-based, interaction, and sensitivity analyses were performed without formal correction for multiple testing, secondary findings should be considered exploratory and hypothesis-generating.

## 6 Conclusion

In this hospital-based cross-sectional study, lower serum 25(OH)D concentration was associated with poorer cognitive performance and higher odds of documented dementia in older adults. These findings should be interpreted as observational associations. Causality cannot be inferred, and reverse causation and residual confounding remain possible. Prospective and interventional studies are needed to clarify whether vitamin D status has independent clinical relevance for cognitive outcomes in hospitalized older adults or primarily reflects broader differences in nutritional, inflammatory, and general health status.

## Data Availability

The datasets analyzed during the current study are available from the corresponding author upon reasonable request. Requests to access these datasets should be directed to Bartłomiej K. Sołtysik bartlomiej.soltysik@umed.lodz.pl.
