# Supplementary material for: Serum 25-hydroxyvitamin D and cognitive performance in hospitalized older adults: a cross-sectional study from Central Poland
Source: Front Nutr. 2026 Aug 13;13:1887375. doi: 10.3389/fnut.2026.1887375 (PMC13518139; doi:10.3389/fnut.2026.1887375)
Supplement: Supplementary file 1 [file Table_1.docx]

Supplementary Material

Throughout the Supplementary Material, MMSE <24 refers to an age- and education-adjusted MMSE score below 24 points.

Table S1. Comparison of included and excluded participants.

| Variable | Included participants (n = 1539) | Excluded participants (n = 109) | p-value |
| --- | --- | --- | --- |
| Age, years | 82 (75–88) | 79 (74–83) | 0.341 |
| BMI, kg/m² | 26.15 (22.77–31.74) | 25.32 (21.88–29.41) | 0.096 |
| Hemoglobin, g/dL | 11.7 (10.2–13.0) | 11.1 (10.1–12.9) | 0.159 |
| GFR, mL/min/1.73 m² | 49.7 (38.2–65.5) | 51.9 (37.6–76.1) | 0.303 |
| CRP, mg/L | 16.8 (3.7–77.5) | 32.2 (6.1–75.6) | 0.102 |
| Albumin, g/L | 34.2 (30.1–38.4) | 33.6 (30.25–36.25) | 0.091 |
| GDS score, points | 5 (3–8) | 4 (2.5–8) | 0.072 |
| Female sex, n (%) | 1057 (68.7%) | 58 (53.2%) | 0.001 |
| Arterial hypertension, n (%) | 1203 (78.2%) | 83 (76.1%) | 0.622 |
| Diabetes mellitus, n (%) | 498 (32.4%) | 41 (37.6%) | 0.258 |
| Coronary artery disease, n (%) | 431 (28.0%) | 33 (30.3%) | 0.611 |
| Atrial fibrillation, n (%) | 477 (31.0%) | 34 (31.2%) | 0.965 |
| Heart failure, n (%) | 1112 (72.3%) | 73 (67.0%) | 0.236 |
| Chronic kidney disease, n (%) | 1024 (66.5%) | 64 (58.7%) | 0.096 |
| Prior stroke, n (%) | 279 (18.1%) | 10 (9.2%) | 0.018 |
| Myocardial infarction, n (%) | 167 (10.9%) | 15 (13.8%) | 0.349 |
| Self-reported vitamin D supplementation, n (%) | 200 (13.0%) | 13 (11.9%) | 0.520 |

Continuous variables are presented as median (interquartile range). Categorical variables are presented as number (%). Between-group comparisons were performed using the Mann–Whitney U test for continuous variables and Pearson’s chi-square test for categorical variables. Valid numbers differed between variables because of missing data. Serum 25(OH)D concentration and cognitive test results were not compared because missing values in these variables were the reasons for exclusion.

Table S2. Diagnostic measures for primary multivariable logistic regression models.

| Outcome | Model χ² | df | p-value | Hosmer–Lemeshow χ² | Hosmer–Lemeshow p-value | Pearson χ²/df | Nagelkerke R² | AUC |
| --- | --- | --- | --- | --- | --- | --- | --- | --- |
| MMSE < 24 | 133.815 | 7 | <0.001 | 9.632 | 0.292 | 0.999 | 0.202 | 0.723 |
| CDT < 6 | 106.210 | 7 | <0.001 | 6.018 | 0.645 | 1.023 | 0.173 | 0.711 |
| Documented dementia | 137.535 | 7 | <0.001 | 7.766 | 0.457 | 1.003 | 0.205 | 0.724 |

Abbreviations: AUC, area under the receiver operating characteristic curve; CDT, Clock Drawing Test; MMSE, Mini-Mental State Examination.

Hosmer–Lemeshow p-values >0.05 indicate no evidence of poor calibration. Pearson χ²/df values close to 1.0 indicate no relevant overdispersion. Collinearity diagnostics did not indicate problematic multicollinearity for serum 25(OH)D; the VIF for serum 25(OH)D was 1.082.

Table S3. Category-based multivariable logistic regression models for adverse cognitive outcomes according to serum 25(OH)D concentration categories.

| Outcome | Serum 25(OH)D category | OR | 95% CI | p-value |
| --- | --- | --- | --- | --- |
| MMSE < 24 | <10 ng/mL | 1.800 | 1.131–2.866 | 0.017 |
|  | 10–19 ng/mL | 1.153 | 0.775–1.715 | 0.520 |
|  | 20–29 ng/mL | 1.178 | 0.765–1.814 | 0.676 |
| CDT < 6 | <10 ng/mL | 1.389 | 0.874–2.208 | 0.159 |
|  | 10–19 ng/mL | 1.218 | 0.816–1.818 | 0.510 |
|  | 20–29 ng/mL | 0.929 | 0.598–1.442 | 0.200 |
| Documented dementia | <10 ng/mL | 2.637 | 1.680–4.140 | <0.001 |
|  | 10–19 ng/mL | 1.403 | 0.945–2.084 | 0.093 |
|  | 20–29 ng/mL | 0.996 | 0.645–1.540 | 0.987 |

Abbreviations: BMI, body mass index; CDT, Clock Drawing Test; CI, confidence interval; GDS, Geriatric Depression Scale; MMSE, Mini-Mental State Examination; OR, odds ratio; 25(OH)D, 25-hydroxyvitamin D.

The reference category was serum 25(OH)D ≥30 ng/mL. Models were adjusted for age, sex, BMI, albumin concentration, GDS score, and prior stroke.

Table S4. Sensitivity analysis after exclusion of participants with prior stroke

| Outcome | Predictor | OR (95% CI) | p-value |
| --- | --- | --- | --- |
| MMSE < 24 | Age, per 1 year | 1.085 (1.061–1.110) | < 0.001 |
|  | BMI, per 1 kg/m² | 0.970 (0.939–1.001) | 0.061 |
|  | Serum 25(OH)D, per 1 ng/mL | 0.983 (0.973–0.994) | 0.003 |
|  | Albumin, per 1 g/L | 0.963 (0.934–0.993) | 0.017 |
|  | GDS score, per 1 point | 1.111 (1.062–1.161) | < 0.001 |
|  | Female sex, yes vs male | 0.935 (0.651–1.341) | 0.714 |
| CDT < 6 | Age, per 1 year | 1.071 (1.047–1.096) | < 0.001 |
|  | BMI, per 1 kg/m² | 0.972 (0.941–1.004) | 0.088 |
|  | Serum 25(OH)D, per 1 ng/mL | 0.989 (0.978–0.999) | 0.034 |
|  | Albumin, per 1 g/L | 0.979 (0.949–1.010) | 0.178 |
|  | GDS score, per 1 point | 1.127 (1.076–1.180) | < 0.001 |
|  | Female sex, yes vs male | 1.300 (0.901–1.877) | 0.161 |
| Documented dementia | Age, per 1 year | 1.087 (1.063–1.112) | < 0.001 |
|  | BMI, per 1 kg/m² | 0.969 (0.938–1.001) | 0.060 |
|  | Serum 25(OH)D, per 1 ng/mL | 0.978 (0.967–0.990) | < 0.001 |
|  | Albumin, per 1 g/L | 0.979 (0.950–1.009) | 0.163 |
|  | GDS score, per 1 point | 1.059 (1.013–1.106) | 0.011 |
|  | Female sex, yes vs male | 0.995 (0.830–1.192) | 0.954 |

Table S5. Sensitivity analysis after exclusion of participants receiving vitamin D3 supplementation

| Outcome | Predictor | OR (95% CI) | p-value |
| --- | --- | --- | --- |
| MMSE < 24 | Age, per 1 year | 1.078 (1.055–1.102) | < 0.001 |
|  | BMI, per 1 kg/m² | 0.966 (0.937–0.997) | 0.030 |
|  | Serum 25(OH)D, per 1 ng/mL | 0.985 (0.974–0.996) | 0.009 |
|  | Albumin, per 1 g/L | 0.950 (0.921–0.980) | 0.001 |
|  | GDS score, per 1 point | 1.116 (1.069–1.165) | < 0.001 |
|  | Female sex, yes vs male | 1.075 (0.758–1.527) | 0.684 |
|  | Prior stroke, yes vs no | 1.336 (0.861–2.072) | 0.196 |
| CDT < 6 | Age, per 1 year | 1.073 (1.050–1.098) | < 0.001 |
|  | BMI, per 1 kg/m² | 0.979 (0.949–1.010) | 0.187 |
|  | Serum 25(OH)D, per 1 ng/mL | 0.989 (0.977–1.000) | 0.048 |
|  | Albumin, per 1 g/L | 0.975 (0.945–1.007) | 0.121 |
|  | GDS score, per 1 point | 1.142 (1.091–1.196) | < 0.001 |
|  | Female sex, yes vs male | 1.260 (0.880–1.803) | 0.207 |
|  | Prior stroke, yes vs no | 1.663 (1.046–2.641) | 0.031 |
| Documented dementia | Age, per 1 year | 1.073 (1.050–1.096) | < 0.001 |
|  | BMI, per 1 kg/m² | 0.961 (0.931–0.991) | 0.012 |
|  | Serum 25(OH)D, per 1 ng/mL | 0.981 (0.970–0.993) | 0.001 |
|  | Albumin, per 1 g/L | 0.973 (0.944–1.002) | 0.069 |
|  | GDS score, per 1 point | 1.071 (1.027–1.117) | 0.001 |
|  | Female sex, yes vs male | 1.111 (0.785–1.573) | 0.552 |
|  | Prior stroke, yes vs no | 1.680 (1.092–2.585) | 0.018 |

Table S6. Sensitivity analysis restricted to participants with normal CRP concentration (≤ 5 mg/L; n = 448)

| Outcome | Predictor | OR (95% CI) | p-value |
| --- | --- | --- | --- |
| MMSE < 24 | Age, per 1 year | 1.068 (1.030–1.108) | <0.001 |
|  | BMI, per 1 kg/m² | 0.935 (0.887–0.986) | 0.013 |
|  | Serum 25(OH)D, per 1 ng/mL | 0.988 (0.974–1.002) | 0.084 |
|  | Albumin, per 1 g/L | 0.946 (0.888–1.008) | 0.084 |
|  | GDS score, per 1 point | 1.105 (1.033–1.182) | 0.004 |
|  | Female sex, yes vs male | 1.184 (0.667–2.102) | 0.564 |
|  | Prior stroke, yes vs no | 1.323 (0.640–2.735) | 0.450 |
| CDT < 6 | Age, per 1 year | 1.032 (1.009–1.056) | 0.007 |
|  | BMI, per 1 kg/m² | 0.971 (0.940–1.003) | 0.071 |
|  | Serum 25(OH)D, per 1 ng/mL | 0.993 (0.984–1.001) | 0.101 |
|  | Albumin, per 1 g/L | 0.995 (0.957–1.035) | 0.821 |
|  | GDS score, per 1 point | 1.085 (1.041–1.132) | <0.001 |
|  | Female sex, yes vs male | 1.144 (0.957–1.368) | 0.138 |
|  | Prior stroke, yes vs no | 1.151 (0.915–1.447) | 0.229 |
| Documented dementia | Age, per 1 year | 1.066 (1.029–1.106) | <0.001 |
|  | BMI, per 1 kg/m² | 0.949 (0.900–0.999) | 0.049 |
|  | Serum 25(OH)D, per 1 ng/mL | 0.989 (0.975–1.003) | 0.121 |
|  | Albumin, per 1 g/L | 0.937 (0.880–0.998) | 0.044 |
|  | GDS score, per 1 point | 1.081 (1.011–1.156) | 0.022 |
|  | Female sex, yes vs male | 1.174 (0.660–2.089) | 0.586 |
|  | Prior stroke, yes vs no | 1.328 (0.646–2.732) | 0.440 |

Table S7. Exploratory multivariable logistic regression models including elevated CRP status and the serum 25(OH)D × elevated CRP interaction term.

| Outcome | Predictor | OR | 95% CI | p-value |
| --- | --- | --- | --- | --- |
| MMSE < 24 | Age, per 1 year | 1.082 | 1.060–1.104 | <0.001 |
|  | BMI, per 1 kg/m² | 0.986 | 0.978–0.995 | 0.002 |
|  | Albumin, per 1 g/L | 0.942 | 0.912–0.974 | <0.001 |
|  | Serum 25(OH)D, per 1 ng/mL | 0.994 | 0.981–1.007 | 0.368 |
|  | GDS score, per 1 point | 1.092 | 1.048–1.138 | <0.001 |
|  | Female sex, yes vs male | 1.054 | 0.755–1.471 | 0.756 |
|  | Prior stroke, yes vs no | 1.418 | 0.923–2.177 | 0.111 |
|  | Elevated CRP >5 mg/L, yes vs no | 1.379 | 0.758–2.510 | 0.293 |
|  | Serum 25(OH)D × elevated CRP status | 0.990 | 0.972–1.009 | 0.317 |
| CDT < 6 | Age, per 1 year | 1.061 | 1.039–1.082 | <0.001 |
|  | BMI, per 1 kg/m² | 0.991 | 0.983–0.999 | 0.021 |
|  | Albumin, per 1 g/L | 0.975 | 0.944–1.008 | 0.141 |
|  | Serum 25(OH)D, per 1 ng/mL | 0.994 | 0.980–1.008 | 0.378 |
|  | GDS score, per 1 point | 1.121 | 1.075–1.169 | <0.001 |
|  | Female sex, yes vs male | 1.220 | 0.873–1.705 | 0.244 |
|  | Prior stroke, yes vs no | 1.709 | 1.105–2.644 | 0.016 |
|  | Elevated CRP >5 mg/L, yes vs no | 1.596 | 0.865–2.944 | 0.134 |
|  | Serum 25(OH)D × elevated CRP status | 0.998 | 0.979–1.018 | 0.868 |
| Documented dementia | Age, per 1 year | 1.086 | 1.064–1.108 | <0.001 |
|  | BMI, per 1 kg/m² | 0.986 | 0.978–0.994 | <0.001 |
|  | Albumin, per 1 g/L | 0.965 | 0.934–0.997 | 0.032 |
|  | Serum 25(OH)D, per 1 ng/mL | 0.989 | 0.975–1.003 | 0.135 |
|  | GDS score, per 1 point | 1.088 | 1.046–1.133 | <0.001 |
|  | Female sex, yes vs male | 1.116 | 0.799–1.558 | 0.520 |
|  | Prior stroke, yes vs no | 1.660 | 1.097–2.511 | 0.016 |
|  | Elevated CRP >5 mg/L, yes vs no | 1.601 | 0.866–2.957 | 0.133 |
|  | Serum 25(OH)D × elevated CRP status | 0.980 | 0.960–1.000 | 0.054 |

Abbreviations: BMI, body mass index; CDT, Clock Drawing Test; CI, confidence interval; CRP, C-reactive protein; GDS, Geriatric Depression Scale; MMSE, Mini-Mental State Examination; OR, odds ratio; 25(OH)D, 25-hydroxyvitamin D.

Elevated CRP status was defined as CRP >5 mg/L. The interaction term was calculated as serum 25(OH)D concentration × elevated CRP status. Models were adjusted for age, sex, BMI, albumin concentration, GDS score, prior stroke, serum 25(OH)D concentration, elevated CRP status, and the interaction term.

Table S8. Extended multivariable logistic regression models for adverse cognitive outcomes including additional comorbidities, nutritional and inflammatory variables.

| Outcome | Predictor | OR | 95% CI | p-value |
| --- | --- | --- | --- | --- |
| MMSE < 24 | Age, per 1 year | 1.086 | 1.062–1.111 | <0.001 |
|  | BMI, per 1 kg/m² | 0.986 | 0.977–0.995 | 0.002 |
|  | Albumin, per 1 g/L | 0.949 | 0.916–0.983 | 0.004 |
|  | Serum 25(OH)D, per 1 ng/mL | 0.988 | 0.979–0.998 | 0.021 |
|  | GDS score, per 1 point | 1.086 | 1.041–1.132 | <0.001 |
|  | NRS score, per 1 point | 1.039 | 0.924–1.168 | 0.523 |
|  | Female sex, yes vs male | 1.061 | 0.747–1.506 | 0.741 |
|  | Prior stroke, yes vs no | 1.505 | 0.971–2.333 | 0.067 |
|  | Elevated CRP >5 mg/L, yes vs no | 1.069 | 0.729–1.567 | 0.733 |
|  | Arterial hypertension, yes vs no | 0.843 | 0.554–1.281 | 0.423 |
|  | Diabetes mellitus, yes vs no | 1.262 | 0.897–1.775 | 0.181 |
|  | Coronary artery disease, yes vs no | 0.606 | 0.413–0.888 | 0.010 |
|  | Atrial fibrillation, yes vs no | 0.922 | 0.648–1.312 | 0.652 |
|  | Heart failure, yes vs no | 1.092 | 0.758–1.574 | 0.635 |
|  | Chronic kidney disease, yes vs no | 0.935 | 0.657–1.329 | 0.707 |
|  | Myocardial infarction, yes vs no | 1.018 | 0.595–1.741 | 0.948 |
| CDT < 6 | Age, per 1 year | 1.073 | 1.049–1.097 | <0.001 |
|  | BMI, per 1 kg/m² | 0.989 | 0.981–0.997 | 0.007 |
|  | Albumin, per 1 g/L | 0.973 | 0.939–1.009 | 0.139 |
|  | Serum 25(OH)D, per 1 ng/mL | 0.993 | 0.983–1.003 | 0.171 |
|  | GDS score, per 1 point | 1.124 | 1.076–1.173 | <0.001 |
|  | NRS score, per 1 point | 0.906 | 0.805–1.020 | 0.103 |
|  | Female sex, yes vs male | 1.279 | 0.898–1.822 | 0.173 |
|  | Prior stroke, yes vs no | 1.779 | 1.134–2.789 | 0.012 |
|  | Elevated CRP >5 mg/L, yes vs no | 1.646 | 1.111–2.438 | 0.013 |
|  | Arterial hypertension, yes vs no | 0.732 | 0.480–1.116 | 0.147 |
|  | Diabetes mellitus, yes vs no | 1.025 | 0.727–1.445 | 0.887 |
|  | Coronary artery disease, yes vs no | 0.928 | 0.630–1.367 | 0.705 |
|  | Atrial fibrillation, yes vs no | 0.701 | 0.489–1.005 | 0.053 |
|  | Heart failure, yes vs no | 1.180 | 0.811–1.717 | 0.387 |
|  | Chronic kidney disease, yes vs no | 0.841 | 0.586–1.206 | 0.347 |
|  | Myocardial infarction, yes vs no | 0.766 | 0.444–1.322 | 0.339 |
| Documented dementia | Age, per 1 year | 1.096 | 1.071–1.121 | <0.001 |
|  | BMI, per 1 kg/m² | 0.985 | 0.978–0.994 | <0.001 |
|  | Albumin, per 1 g/L | 0.958 | 0.925–0.992 | 0.017 |
|  | Serum 25(OH)D, per 1 ng/mL | 0.978 | 0.968–0.989 | <0.001 |
|  | GDS score, per 1 point | 1.088 | 1.044–1.133 | <0.001 |
|  | NRS score, per 1 point | 0.911 | 0.810–1.024 | 0.118 |
|  | Female sex, yes vs male | 1.102 | 0.776–1.567 | 0.587 |
|  | Prior stroke, yes vs no | 1.791 | 1.170–2.740 | 0.007 |
|  | Elevated CRP >5 mg/L, yes vs no | 0.950 | 0.645–1.399 | 0.796 |
|  | Arterial hypertension, yes vs no | 0.652 | 0.431–0.987 | 0.043 |
|  | Diabetes mellitus, yes vs no | 1.032 | 0.736–1.448 | 0.853 |
|  | Coronary artery disease, yes vs no | 0.687 | 0.468–1.008 | 0.055 |
|  | Atrial fibrillation, yes vs no | 0.836 | 0.589–1.186 | 0.315 |
|  | Heart failure, yes vs no | 0.894 | 0.619–1.291 | 0.549 |
|  | Chronic kidney disease, yes vs no | 0.963 | 0.674–1.375 | 0.834 |
|  | Myocardial infarction, yes vs no | 0.937 | 0.543–1.614 | 0.813 |

Abbreviations: AF, atrial fibrillation; BMI, body mass index; CAD, coronary artery disease; CDT, Clock Drawing Test; CI, confidence interval; CKD, chronic kidney disease; CRP, C-reactive protein; DM, diabetes mellitus; GDS, Geriatric Depression Scale; HF, heart failure; MI, myocardial infarction; MMSE, Mini-Mental State Examination; NRS, Nutritional Risk Screening; OR, odds ratio; 25(OH)D, 25-hydroxyvitamin D.

All models included age, sex, BMI, albumin concentration, serum 25(OH)D concentration, GDS score, NRS score, prior stroke, elevated CRP status >5 mg/L, arterial hypertension, diabetes mellitus, coronary artery disease, atrial fibrillation, heart failure, chronic kidney disease, and myocardial infarction. Binary variables are presented as yes vs no. For CDT < 6, odds ratios were recalculated so that all estimates refer to the adverse cognitive outcome.

Table S9. Formal tests of interaction between serum 25(OH)D concentration and sex for adverse cognitive outcomes.

| **Outcome** | **Interaction term** | **OR** | **95% CI** | **p-value** |
| --- | --- | --- | --- | --- |
| MMSE < 24 | Serum 25(OH)D × male sex | 1.000 | 0.980–1.022 | 0.966 |
| CDT < 6 | Serum 25(OH)D × male sex | 0.988 | 0.968–1.008 | 0.239 |
| Documented dementia | Serum 25(OH)D × male sex | 1.024 | 0.997–1.052 | 0.081 |

Abbreviations: BMI, body mass index; CDT, Clock Drawing Test; CI, confidence interval; GDS, Geriatric Depression Scale; MMSE, Mini-Mental State Examination; OR, odds ratio; 25(OH)D, 25-hydroxyvitamin D.

The interaction term was calculated as serum 25(OH)D concentration (per 1 ng/mL) × male sex (male = 1, female = 0). Models included age, BMI, albumin concentration, GDS score, prior stroke, serum 25(OH)D concentration, sex, and the interaction term. All odds ratios were oriented so that estimates refer to the adverse cognitive outcome.
